# Transforming through democracy, human rights, and sustainable development at the 5th National Conference on Workers’ Health

**DOI:** 10.1590/S2237-96222025v34e20250669.en

**Published:** 2025-10-20

**Authors:** Ethel Leonor Maciel, Luis Leão, Luciene de Aguiar Dias, Douglas Oliveira Carmo Lima, Isabella Maio

**Affiliations:** 1 Universidade Federal do Espírito Santo, Vitória, ES, Brazil; 2 Ministério da Saúde, Departamento de Vigilância em Saúde Ambiental e Saúde do Trabalhador, Brasília, DF, Brazil; 3 Fundação Oswaldo Cruz, Rio de Janeiro, RJ, Brazil; 4 Ministério da Saúde, Secretaria de Vigilância em Saúde e Ambiente, Brasília, DF, Brazil

Convened by the National Health Council under the guiding principles of the Brazilian Unified Health System (*Sistema Único de Saúde* - SUS), the nationwide conferences on workers’ health serve as democratic arenas for debate, collective development, and the shaping of public policies across and within sectors, which are essential for Brazil’s working population. Since the first edition in 1986, the discussions at these conferences have reflected the tensions between models of economic development and the guarantee of the right to life and health for the working population. 

The field of workers’ health is not merely a technical or scientific issue; it is a field of political dispute, reflecting the contradictions of a society that has historically prioritized production over life. Scheduled to culminate in its national assembly in August 2025, the 5th Workers’ Health Conference is already underway across Brazil. It emerges in a context of renewed urgencies: new realities in the world of work, accelerated precarization, in addition to the impacts of the COVID-19 pandemic and the climate crisis on the lives of the working class (1) The relevance of the 5th Conference is best understood within the historical context of its predecessors (2,3), each distinguished by collective struggles and victories.

Convened in the same year as the 8th National Health Conference in the context of Brazil’s democratic transition, the inaugural Workers’ Health Conference was a pivotal moment for the field. It facilitated crucial debates that ultimately led to the incorporation of workers’ health into the 1988 Citizen Constitution (4). It is no coincidence that workers’ health actions are listed in Article 200 as one of the core responsibilities of SUS; these actions were later further elaborated in Law No. 8,080/1990 (5). This edition created a platform to denounce work-related diseases and accidents, exposure to toxic substances, the rampant use of pesticides, as well as the invisibility of occupational diseases, proposing the creation of a list of work-related diseases for compulsory notification.

In this context, the 1st National Workers’ Health Conference fostered the participation and significant contributions of academics, technicians, and intellectuals from trade unions and social movements. The penalization of the capital in cases of work-related diseases and deaths was explicit and marked the renewed defense of the lives and health of Brazilian workers (6).

Amid the neoliberal advance of the 1990s, the 2nd National Workers’ Health Conference highlighted the deleterious effects of productive restructuring: outsourcing, unemployment, and the dismantling of the Brazilian Consolidation of Labor Laws (*Consolidação das Leis do Trabalho* - CLT). This edition also sounded the alarm on the emergence of new forms of work-related disease, such as repetitive strain injuries and musculoskeletal disorders; reinforced the importance of health surveillance and, above all, Workers’ Health Surveillance as a tool for monitoring and evaluation; and generated data that would later guide the construction of workers’ health policy. 

Under the motto “Working, Yes! Getting Sick, No!”, the 3rd National Workers’ Health Conference, held in 2005, addressed the impacts of globalization, including transnational precaritization and environmental risks. This conference broadened the debate to include rural workers, informal workers, and communities affected by pesticides. It revived discussions on formulating the National Workers’ Health Policy (*Política Nacional de Saúde do Trabalhador e da Trabalhadora* - PNSTT), which would be published seven years later. It advocated for intersectorality as the path forward for public policies.

In 2014, in a Brazil fragmented by protests and economic crisis, the 4th Conference emphasized the defense of SUS and the fight against the rollback of rights. It addressed issues such as mental health in the workplace, moral harassment, and the challenges posed by nanotechnology. It also placed the effective implementation of the PNSTT and the National Network for Comprehensive Workers’ Healthcare (*Rede Nacional de Atenção Integral à Saúde do Trabalhador e da Trabalhadora* - RENAST) within SUS on the agenda. The most important legacy of this conference was its unwavering defense of social participation at a time when conservative influence was on the rise.

Despite being fundamental spaces for reflection and social participation, the last three conferences yielded minimal progress in the face of the challenges confronting workers’ health in Brazil. Their resolutions and recommendations failed to ensure the effective implementation of public policy and the corresponding network within SUS. The conferences were characterized by ideological clashes with historically conservative views within occupational medicine and health, which persist in proposing measures that merely mitigate work-related diseases, rather than prevent them at their source.

In a significant departure from this conservative viewpoint, a major advancement occurred in 2023 with the publication of a new List of Work-Related Diseases, leading to their formal regulation and recognition, as well as the identification of new forms of occupational diseases (7). Moreover, it highlighted the critical importance of surveillance systems in gathering data on diseases associated with pesticide exposure. In addition, the 100.0% increase in funding for the entire National Network for Comprehensive Workers’ Health Care was fundamental (8) for strengthening these actions.

Within the context of this publication and a renewed perspective on the List of Work-Related Diseases, the 5th National Conference on Workers’ Health, after ten years, seeks to break with this paradigm by adopting the theme “Workers’ Health as a Human Right” as its central focus. In addition to reflecting the complexity of the current historical moment, this theme proposes a bold epistemological shift in the field by aiming to elevate the legal, institutional, social, and symbolic status of workers’ health in Brazil (8). Therefore, the conference will discuss long-standing and emerging challenges in the field through the lens of human rights, amidst the ongoing reconstruction of a country that has recently emerged from a political project defined by the denial of these fundamental rights.

The proposals and discussions at the 5th Conference will be structured around the following core themes: The National Workers’ Health Policy; New forms of labor relations and their impact on workers’ health; Popular participation in workers’ health for effective social control (9). Each of these themes considers the persistent contradictions within the field, such as the failure to implement the PNSTT within SUS fully; the weakness of Workers’ Health Surveillance actions in addressing and preventing harms and diseases stemming from new, precarious forms of work; the vulnerability of meaningful social participation in both the PNSTT and the development of Workers’ Health Surveillance actions.

The debate on democracy, human rights, and sustainable development cuts across all thematic axes, based on the understanding that: there is no health without democracy; human rights are essential for every citizen; and any form of development will remain unsustainable as long as it is disconnected from improvements in working and health conditions (10,11,12).

Workers’ Health Conferences are spaces of memory and projection, of reclamation and political imagination. These conferences enable the Brazilian working population to contribute to the formulation of public policy and guide the actions of the State across all levels of the federation toward a decentralized and integrated health system (13). 

If the 1st Conference buried the authoritarian logic of the dictatorship, the 5th must respond to the threat of emerging fascisms, to machismo, racism, ableism, the individualizing and wealth- and land-concentrating neoliberal project, and the commodification of life. The conference can pave new paths for collective actions aimed at the structural transformation of society and the economy. It must prioritize the fight against labor precarity, recognize land as a fundamental determinant of health, and combat inequalities in the world of work, violence, dispossession, exploitation, and the oppression of working populations, among other critical issues.

Data from the Observatory of Safety and Health at Work (from the Labor Prosecution Service and the International Labor Organization) revealed that between 2012 and 2023, counting only formal workers with signed work permits, there were over 7 million workplace accidents, with an estimated 2 million additional underreported cases in the country. In the same period, 27,554 deaths were recorded, caused by and at work (14). 

In light of the data, it is clear that a serious public health issue is affecting the lives of Brazilian men and women. It is up to the 5th National Conference on Workers’ Health to deliver strong responses to this public health crisis and to set forth strategic, intersectoral, and participatory actions to put an end to workers’ morbidity and mortality. The goal is to strengthen the field in the coming years, including by giving greater visibility to workers’ health within the structures of the Brazilian National Health System (SUS), as the conference will enable broader understanding and effective implementation of the National Policy on Workers’ Health (PNSTT) beyond the Brazilian National Network for Comprehensive Workers’ Health Care, reaching the SUS network as a whole. 

The 5th Conference will also serve as an opportunity to debate expanding the scope of action of the General Coordination for Workers’ Health and, consequently, the need for its recognition as the coordinating body for protecting workers’ lives within the structure of the Brazilian Ministry of Health, given that the PNSTT encompasses more than just Occupational Health Surveillance. 

Ultimately, we recall the enduring words of public health advocate Sérgio Arouca: “There is no health without democracy.” This means fighting for social justice on a planet in collapse—a planet that disregards the central role of work in human life. By framing workers’ health as a human right, the 5th Conference not only restores this centrality but also establishes work itself as a fundamental and inalienable right. The 5th National Workers’ Health Conference, therefore, is a call to action: only through collective mobilization can we ensure that no worker is left behind.
