# Supplementary material for: Transforming through democracy, human rights, and sustainable development at the 5th National Conference on Workers’ Health
Source: Epidemiol Serv Saude. 2025 Oct 20;34:e20250669. doi: 10.1590/S2237-96222025v34e20250669.en (PMC12536955; doi:10.1590/S2237-96222025v34e20250669.en)
Supplement: Translation

# Translation: Transformar com democracia, direitos humanos e desenvolvimento sustentável, na 5ª Conferência Nacional de Saúde do Trabalhador e da Trabalhadora

**DOI:** 10.1590/S2237-96222025v34e20250669.pt

**Authors:** Ethel Leonor Maciel, Luis Leão, Luciene de Aguiar Dias, Douglas Oliveira Carmo Lima, Isabella Maio

**Affiliations:** 1 Universidade Federal do Espírito Santo, Vitória, ES, Brasil; 2 Ministério da Saúde, Departamento de Vigilância em Saúde Ambiental e Saúde do Trabalhador, Brasília, DF, Brasil; 3 Fundação Oswaldo Cruz, Rio de Janeiro, RJ, Brasil; 4 Ministério da Saúde, Secretaria de Vigilância em Saúde e Ambiente, Brasília, DF, Brasil

As conferências nacionais de saúde do trabalhador, organizadas sob os princípios do Sistema Único de Saúde (SUS) pelo Conselho Nacional de Saúde, são espaços democráticos de debate, construção coletiva e formulação de políticas públicas intra e intersetoriais, fundamentais para a população trabalhadora do Brasil. Desde a primeira edição, em 1986, as discussões presentes nas conferências refletem as tensões entre os modelos de desenvolvimento econômico e a garantia de direitos à vida e à saúde da população trabalhadora. 

O campo da saúde do trabalhador e da trabalhadora não é apenas uma questão técnica ou científica; é um campo de disputa política, reflexo das contradições de uma sociedade que historicamente priorizou a produção sobre a vida. A 5ª Conferência Nacional de Saúde do Trabalhador e da Trabalhadora, que já está acontecendo em todo o Brasil e terá a etapa nacional em agosto de 2025, emerge em um contexto de urgências renovadas: as novas relações no mundo do trabalho, a precarização acelerada, além dos impactos da pandemia de covid-19 e da crise climática na vida da classe trabalhadora (1). Para entender a relevância da 5ª Conferência, é preciso relembrar as conferências anteriores (2,3), cada uma delas marcada por lutas e conquistas coletivas.

Realizada no mesmo ano da 8ª Conferência Nacional de Saúde, na conjuntura da redemocratização, a 1ª Conferência Nacional de Saúde do Trabalhador foi um marco para o campo e possibilitou reflexões importantes que contribuíram para a inserção da saúde do trabalhador na Constituição Cidadã de 1988 (4). Não à toa, as ações de saúde do trabalhador aparecem no artigo 200 como uma das competências do SUS; posteriormente, as ações foram mais bem aprofundadas na Lei nº 8.080/1990 (5). Essa Conferência possibilitou denunciar adoecimentos e acidentes relacionados ao trabalho, exposição a substâncias tóxicas, uso desenfreado de agrotóxicos, além da invisibilidade das doenças ocupacionais, propondo a construção de uma lista de doenças relacionadas ao trabalho para a notificação compulsória.

Nesse contexto, a 1ª Conferência Nacional de Saúde do Trabalhador propiciou a participação e os avanços significativos de acadêmicos, técnicos e intelectuais orgânicos oriundos do movimento sindical e de movimentos sociais. A penalização ao capital em casos de adoecimentos e mortes dos trabalhadores relacionados ao trabalho era explícita e marcou a retomada da defesa da vida e da saúde dos trabalhadores brasileiros (6).

Em meio ao avanço neoliberal dos anos 1990, a 2ª Conferência Nacional de Saúde do Trabalhador destacou os efeitos deletérios da reestruturação produtiva: terceirização, desemprego e desmonte da Consolidação das Lei do Trabalho. Essa Conferência também alertou para o surgimento de novas formas de adoecimento, como as lesões por esforço repetitivo e os distúrbios osteomusculares relacionado ao trabalho; reforçou a importância da vigilância em saúde e, sobretudo, da Vigilância em Saúde do Trabalhador como instrumento de monitoramento e avaliação; e possibilitou dados que puderam posteriormente guiar a construção da política de saúde do trabalhador. 

Sob o lema “Trabalhar, Sim! Adoecer, Não!”, a 3ª Conferência Nacional de Saúde do Trabalhador, realizada em 2005, confrontou os impactos da globalização, como a precarização transnacional e os riscos ambientais. Essa Conferência ampliou o debate para trabalhadores rurais, informais e comunidades atingidas por agrotóxicos, retomou a discussão da construção da Política Nacional de Saúde do Trabalhador e da Trabalhadora (PNSTT), que seria publicada sete anos depois, e defendeu a intersetorialidade como caminho para as políticas públicas.

Em 2014, em um Brasil fragmentado por protestos e crise econômica, a 4ª Conferência enfatizou a defesa do SUS e a luta contra o retrocesso de direitos. Abordou temas como a saúde mental no trabalho, o assédio moral e os desafios da nanotecnologia e pautou a efetivação da PNSTT e da Rede Nacional de Atenção Integral à Saúde do Trabalhador e da Trabalhadora no SUS. O maior legado dessa Conferência foi a defesa intransigente da participação social em um momento de ascensão conservadora.

Apesar de constituírem espaços fundamentais de reflexão e participação social, as três últimas conferências contaram com avanços muito limitados diante dos desafios postos para a saúde dos trabalhadores e trabalhadoras no Brasil. Os encaminhamentos não garantiram a efetivação da política pública e da rede no SUS. As conferências foram marcadas por disputas com visões historicamente conservadoras da medicina do trabalho e da saúde ocupacional, que insistem em propor ações mitigadoras do adoecimento relacionado ao trabalho.

Em uma inflexão sobre essa visão conservadora, em 2023, ocorreu importante avanço na regulamentação e no reconhecimento das doenças relacionadas ao trabalho com a publicação da Lista de Doenças Relacionadas ao Trabalho. Esta apontou novas formas de adoecimento relacionadas ao trabalho (7) e a importância da vigilância para a captação de informação de doenças relacionadas ao uso de agrotóxicos. Além disso, a majoração dos recursos em 100,0% em toda a Rede Nacional de Atenção Integral à Saúde do Trabalhador e da Trabalhadora foi fundamental (8) para o fortalecimento dessas ações.

É no contexto dessa publicação e de um novo olhar para a Lista de Doenças Relacionadas ao Trabalho que, após dez anos, a 5ª Conferência Nacional de Saúde do Trabalhador e da Trabalhadora busca romper com esse paradigma ao trazer como tema central a “Saúde do Trabalhador e da Trabalhadora como Direito Humano”. Além de refletir a complexidade do momento histórico, esse tema propõe ousado giro epistemológico para o campo ao propor a elevação do patamar jurídico, institucional, social e simbólico da saúde do trabalhador e da trabalhadora no Brasil (8). Portanto, a conferência discutirá antigos e novos desafios do campo, sob a luz dos direitos humanos, no contexto de reconstrução de um país recém-saído de um projeto de negação desses direitos fundamentais.

Na 5ª Conferência, as propostas serão pautadas nos seguintes eixos: Política Nacional de Saúde do Trabalhador e da Trabalhadora; novas relações de trabalho e saúde do trabalhador e da trabalhadora; e participação popular na saúde dos trabalhadores e das trabalhadoras para o controle social (9). Cada um dos eixos considera as contradições ainda presentes no campo com, por exemplo, a não efetivação da PNSTT no SUS; as fragilidades das ações de Vigilância em Saúde do Trabalhador para responder e prevenir os agravos e adoecimentos relacionados às novas relações de trabalho precarizadas; e a vulnerabilidade do envolvimento e da efetiva participação social na PNSTT e na construção das ações de Vigilância em Saúde do Trabalhador.

O debate sobre democracia, direitos humanos e desenvolvimento sustentável é transversal a todos os eixos, considerando que: não há saúde sem democracia; os direitos humanos são primordiais para todo e qualquer cidadão; e qualquer desenvolvimento continuará sendo insustentável, enquanto estiver apartado da melhoria das condições de trabalho e saúde (10,11,12).

As conferências de saúde do trabalhador e da trabalhadora são espaços de memória e projeção, resgate e imaginação política. Essas conferências permitem que a população trabalhadora brasileira contribua para a formulação de políticas públicas e direcione as ações do Estado, em todas as esferas da Federação, para o sistema descentralizado e integrado de saúde (13). 

Se a 1ª Conferência enterrou a lógica autoritária da ditadura, a 5ª precisa responder ao risco de novos fascismos, ao machismo, ao racismo, ao capacitismo, ao projeto neoliberal individualizante e concentrador de terra e renda e à mercantilização da vida.

A conferência pode abrir novos caminhos para ações coletivas de transformação estrutural da sociedade e economia, pautando a desprecarização do trabalho, a terra como elemento determinante da saúde e o combate às desigualdades no mundo do trabalho, às violências, às expropriações, à exploração e à opressão das populações trabalhadoras etc.

Os dados do Observatório de Segurança e Saúde no Trabalho do Ministério Público do Trabalho e da Organização Internacional do Trabalho revelaram que, entre 2012 e 2023, contabilizando apenas trabalhadores formais com carteira assinada, ocorreram mais de 7 milhões de acidentes de trabalho e foram estimados mais de 2 milhões de casos subnotificados no país. No mesmo período, foram contabilizadas 27.554 mortes no e pelo trabalho (14). 

Diante dos dados, nota-se o grave problema de saúde pública que assola a vida dos brasileiros e brasileiras. Cabe à 5ª Conferência Nacional de Saúde do Trabalhador e da Trabalhadora dar respostas contundentes ao drama sanitário brasileiro e pautar ações estratégicas, intersetoriais e participativas, para acabar com a morbimortalidade de trabalhadores. Tem-se como intuito o fortalecimento do campo para os próximos anos, inclusive com mais visibilidade à saúde do trabalhador e da trabalhadora nas estruturas do SUS, já que a conferência possibilitará a ampliação do entendimento e a efetiva implementação da PNSTT para além da Rede Nacional de Atenção Integral à Saúde do Trabalhador e da Trabalhadora, alcançando a rede SUS. 

A 5ª Conferência será também uma oportunidade para debater a ampliação da dimensão de atuação da Coordenação Geral de Saúde do Trabalhador e, consequentemente, da necessidade de seu reconhecimento como instância articuladora do cuidado com a vida dos trabalhadores e trabalhadoras do Brasil na estrutura do Ministério da Saúde, considerando que a PNSTT não prevê unicamente a Vigilância em Saúde do Trabalhador. 

Por fim, como bem lembrava o sanitarista Sérgio Arouca, “a saúde se faz com democracia”. Isso significa lutar por justiça social em um planeta em colapso e que desconsidera a centralidade do trabalho na vida humana. A saúde do trabalhador como direito humano não só retoma essa centralidade como também pauta o trabalho como direito fundamental e inalienável. A 5ª Conferência Nacional de Saúde do Trabalhador e da Trabalhadora é, portanto, um chamado à ação: somente a mobilização coletiva garantirá que nenhum trabalhador ou trabalhadora seja esquecido.
